# Bouncing of Hydroxylated Silica Nanoparticles: an Atomistic Study Based on REAX Potentials

**DOI:** 10.1186/s11671-020-03296-y

**Published:** 2020-03-30

**Authors:** Maureen L. Nietiadi, Yudi Rosandi, Herbert M. Urbassek

**Affiliations:** 1 Physics Department and Research Center OPTIMAS, University Kaiserslautern, Erwin-Schrödinger-Straße, Kaiserslautern, 67663 Germany; 2 Department of Geophysics, Universitas Padjadjaran, Jatinangor, Sumedang, 45363 Indonesia

**Keywords:** Silica, Hydroxylation, Cluster collisions, Molecular dynamics, Nanoparticles

## Abstract

Clean silica surfaces have a high surface energy. In consequence, colliding silica nanoparticles will stick rather than bounce over a wide range of collision velocities. Often, however, silica surfaces are passivated by adsorbates, in particular water, which considerably reduce the surface energy. We study the effect of surface hydroxylation on silica nanoparticle collisions by atomistic simulation, using the REAX potential that allows for bond breaking and formation. We find that the bouncing velocity is reduced by more than an order of magnitude compared to clean nanoparticle collisions.

## Background

Collisions of silica nanoparticles (NPs) play an important role in many branches of geophysics and planetary sciences. Examples are provided by the physics of sand dunes on Earth and other planets [1]; in the protoplanetary disks around newborn stars, collisions between silica particles constitute the first process on the stage to planet formation [2, 3]; in mature solar systems, sources of dust particles are available by emission from comets [4, 5] or asteroid collisions [6–8]. In addition, silica NP collisions also serve as the prototypical example of granular mechanics and hence are used in experiments aimed at understanding the physics of NP collisions [9–12]. Usually, the material parameters describing silica grains are taken from experimental data. Here, bulk properties such as the elastic moduli are subject to little error, while surface properties—in particular, the specific surface energy—are prone to considerable fluctuations between individual experiments [13]. The surface energy enters important quantities such as the probability of sticking and the energy transfer during collisions.

These fluctuations are induced by adsorbates, in particular water, that are commonly present on the grain surface. Since silica is a polar material, adsorbates or their dissociation products may passivate dangling bonds at the grain surface, thus strongly altering the surface energy.

Atomistic simulations based on the interatomic interactions aim at assisting the understanding of NP collision processes. This approach is non-trivial in the case of polar materials such as silica, where adsorbates may strongly influence interactions at the surface [14]. Thus, it has been found that when modeling silica collisions with clean surfaces, the colliding NPs stick rather than bounce over a wide range of velocities [15], in contrast to experiments [16–18]. One may presume [13] that the surface passivation by water—that is, the surface hydroxylation—is responsible for this behavior. Up to now, simulations of passivated silica NP collisions could be performed only with small (≤ 2 nm) grains [19], which show a considerable spread in collisional outcomes due to their nanoscopic size.

We want to demonstrate here the effect of surface hydroxylation on silica NP collisions by directly comparing the results of hydroxylated NPs with those obtained previously for clean silica [15]. This will allow us to conclude that the effect of hydroxylation on collisional properties can be captured by a decreased specific surface energy.

## Method

We use the REAX potential to model the interatomic interactions between Si, O, and H [20]. While this potential is more than a factor of 100 slower than pair potentials, it has the benefit to include van der Waals and electrostatic interactions as well as covalent bonds within one framework and therefore belongs to the most advanced interatomic potentials available [21]. It has been designed with the special purpose of including chemical reactions into classical MD simulations and thus to help bridge the gap between classical and quantum simulations [22]. Therefore, this potential allows us to capture the bond breakings and formations that may occur during high-energy collisions both in silica and in the hydroxylated surface layer. In addition, it permits to model the hydroxylation process, i.e., the O–H and the OH–Si interaction.

To produce the NPs, we make use of the amorphous silica that was prepared recently [15] according to the quenching procedure described by Huff et al. [23]. This silica block was relaxed in the REAX potential and spheres with radii of *R*=10, 15, and 20 nm—containing 0.32, 1.01, and 2.38 million atoms per sphere, respectively—were cut from this sample and relaxed to the final temperature of 200 K. This temperature was chosen as it is typical for the asteroid belt in the solar system; however, it is not expected that the temperature will strongly change the collision dynamics as long as it is below the temperature where the hydroxylation layer starts dissolving (460 K) [15, 24].

Surface hydroxylation is performed similar to previous studies on the silica—water interface [19, 25–28]. We identify under-coordinated O and Si atoms at the NP surface; H is added to under-coordinated O and OH to under-coordinated Si; in both cases, thus terminal silanol groups (–SiOH) are created. The hydroxylated NP is then allowed to relax using a conjugate gradient algorithm. The area density of the silanol groups created at the surface amounts to 4.12 (4.93, 4.89) nm ^−2^ for the *R*=10 (15, 20) nm sphere. Experimental silanol densities are in the range of 2.6–4.6 OH nm ^−2^ [24]. Other simulation studies gave lower values, 2.0–2.5 OH/nm^2^ [25, 27] or even only 1.3–1.8 OH/nm^2^ [19], but also higher values of 6.6 OH/nm^2^ [26, 28]. This large variety is due to (i) possibly incomplete identification of under-coordinated surface atoms and (ii) atomic surface roughness resulting in a larger effective surface area than the calculated area [26, 28]. Note that in the experiment, the silanol concentration also depends on the pH value of the environment [14, 29, 30]. We conclude that our silanol surface densities are not unrealistic.

**Fig. 1:**
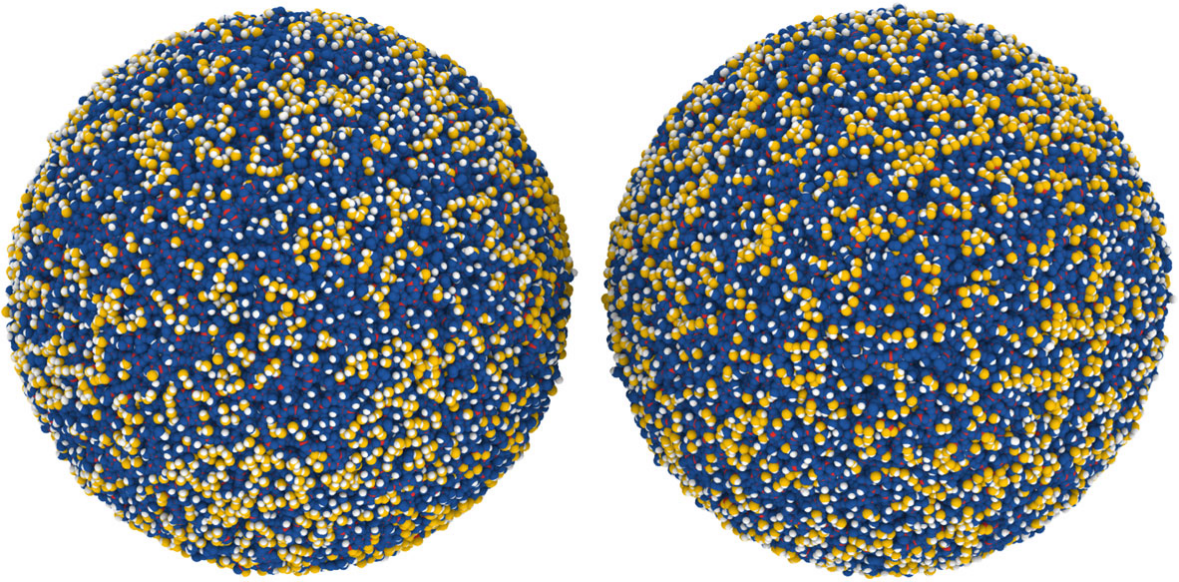
Hydroxylated silica NPs. Hydroxylated silica NPs of radius *R*=10 nm immediately before collision. Red, Si; blue, O bonded to Si; orange, O in an OH group; white, H

The collision is started by duplicating the NP and rotating the two NPs randomly with respect to each other, see Fig. 1. For each collision system, 5 simulations are performed to gather statistics; the simulations differ from each other by the rotation angles. The two copies are then shot onto each other with a relative velocity *v*. Only the central collisions are considered. The simulations are run until the final fate—bouncing or sticking—could be clarified, see Refs. [15, 31] for details.

The molecular dynamics simulations are performed with the LAMMPS code [32]. Atomic snapshots are generated with OVITO [33]. The simulation of a single collision—for the example of *R*=20 nm spheres colliding with *v*=100 m/s—took around 120 h on 256 cores.

## Results and Discussion

The coefficient of restitution (COR) measures the relative velocity of the NPs after collision, *v*^′^, relative to that before collision, *v*, as:

1 $$ e = \frac{|v^{\prime}|}{|v|}.  $$

**Fig. 2:**
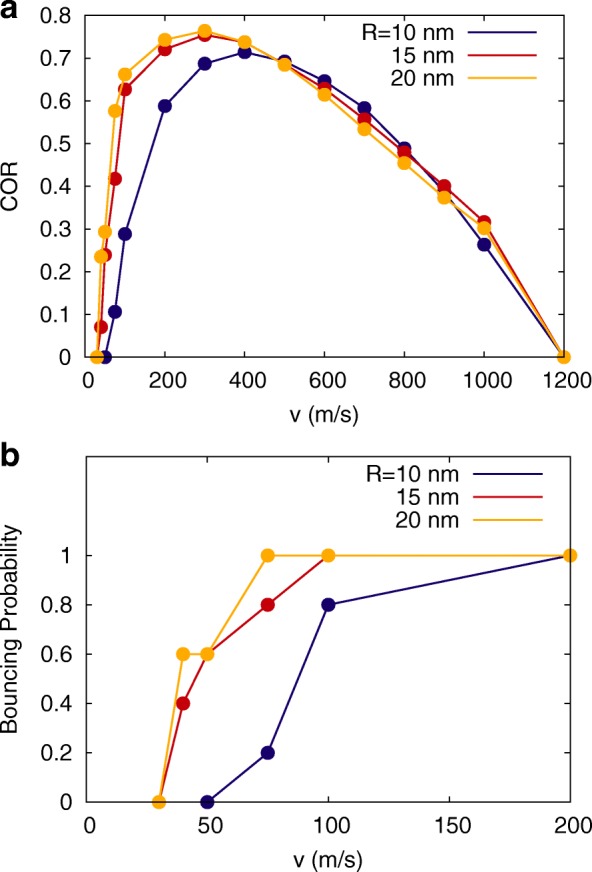
Coefficient of restitution and bouncing probability. **a** Coefficient of restitution (COR) and **b** bouncing probability as a function of impact velocity, *v*, for various NP radii *R*. Symbols, simulation results. Lines are to guide the eye

It vanishes when the two NPs stick; a non-vanishing COR can hence be taken as a sign of bouncing. Figure 2a shows the COR as measured in our simulations. Hydroxylated NPs stick at low velocities, bounce in a large bouncing window [31] up to velocities of 1200 m/s, and then stick again. Sticking at large velocities is caused by strong NP deformations that dissipate energy and let the colliding grains fuse together.

Sticking at low velocities, on the other hand, is caused by the adhesive forces between the NPs. We plot the bouncing probability, *p*_*b*_, that is the fraction of collisions leading to bouncing in our simulations, at low collision velocities in Fig. 2b. We identify the bouncing velocity as the smallest velocity where the bouncing probability—and hence the COR—are non-zero. Note that the range of velocities, in which 0<*p*_*b*_<1—i.e., where the relative orientation of the two colliding spheres decides on the collision outcome—considerably widens for smaller NPs. Thus, sticking is certain for *v* ≥ 75 m/s for large spheres (*R*=20 nm), while it is certain only for velocities reaching at least 200 m/s for *R*=10 nm spheres.

The macroscopic Johnson-Kendall-Roberts (JKR) [34] theory of adhesive contacts predicts the bouncing velocity *v*_*b*_ as [35–37]:

2 $$ v_{b} = \left(\frac {C} {\rho} \right)^{1/2} \left(\frac {\gamma^{5}} {E_{\text{ind}}^{2} R^{5}} \right)^{1/6}.  $$

This law holds for collisions of two NPs with the same radius *R*, mass density *ρ*, specific surface energy *γ*, and an elastic modulus *E*_ind_, which is determined as *E*_ind_=*E*/(1−*ν*^2^) from Young’s modulus *E* and the Poisson ratio *ν*. In our previous study of silica collisions [15], we determined *ρ*=2.25 g/cm^3^ and *E*_ind_=67.1 GPa; we shall use these values also here. The constant *C* is subject to quite some uncertainty, since it includes the effect of energy dissipation channels—such as defect formation in the material and excitation of oscillations—which are not easily assessed [9, 13, 36–38]. The most recent experimental estimate was obtained from collision experiments with silica spheres of radius 250 and 600 nm and gave *C*=57.9 [13]. However, our simulation data for clean silica spheres of radius ≤ 25 nm [15] need a value of *C*=669 for fitting. This high value is caused in particular by the formation of a dynamic adhesive neck between the NPs during the collision.

The specific surface energy *γ* is not easily calculated for our hydroxylated NPs due to the inhomogeneity of the system. For clean silica surface, we determined *γ*=1.43±0.09 J/m^2^ [15]. This value is in approximate agreement with experimental data of clean silica [13].

**Fig. 3:**
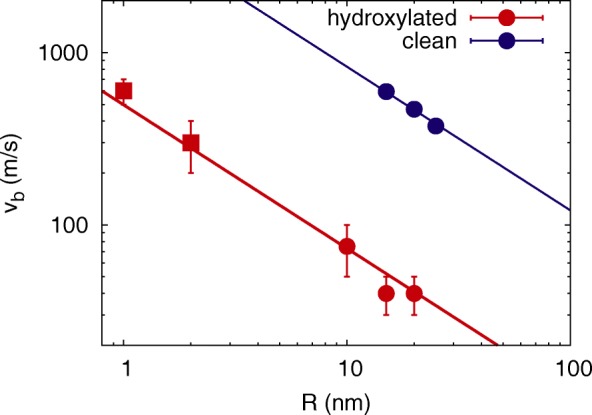
Bouncing velocity. Dependence of the bouncing velocity, *v*_*b*_, of hydroxylated silica NPs on the NP radius, *R*. Present (circle) and previous (square) [19] simulation data of hydroxylated silica spheres compared to simulation data of clean silica spheres [15]. Symbols, simulation results. Line: Eq. 2. The error bars of the clean NPs are smaller than the symbol size

However, the specific surface energy *γ* may be obtained from a fit of Eq. 2 to our simulation data. Figure 3 demonstrates that our data indeed agree well with the *R*^−5/6^ dependence of the JKR theory. The fit of the data to Eq. 2 gives a value of *γ*=0.078 J/m^2^, i.e., a reduction by a factor of almost 20 with respect to the clean silica results. Such a low value the surface energy is in good agreement with the range of data observed experimentally in silica on which water has been adsorbed and where values in a range of *γ*=0.02–0.3 J/m^2^ have been reported [13, 24].

Quadery et al. [19] simulated nanometer-sized hydroxylated a-SiO_2_ NPs (radii of 1 and 2 nm) with the REAX potential and demonstrated that such NPs exhibit reduced adhesion. They report bouncing velocities of around 0.6 and 0.3 km/s for *R*=1 and 2 nm, respectively. These NPs had a quite an irregular surface structure, probably because they were created by melting and quenching the NP in vacuum, rather than cutting them out from a large a-SiO_2_ sample. When including these data into our *v*_*b*_(*R*) diagram, Fig. 3, we observe that these previous data align well with our results and are described by Eq. 2 with the same parameters.

Note that clean, i.e., not hydroxylated, silica NPs bounce at considerably higher velocities, cf. Fig. 3. Their bouncing velocities are again described with Eq. 2, if only the value of *γ* is adapted to 1.43 J/m^2^, see the line in Fig. 3. These collisions were simulated in [15] using the Munetoh potential [39] for silica. We verified that for the REAX potential, similar results are obtained. In particular, for the *R*=20 nm NP, we obtained a bouncing velocity of 475 m/s in close agreement with the previous result of 469 m/s [15], while for *R*=15 nm NPs, no bouncing was observed in the velocity range of 300–800 m/s.

**Fig. 4:**
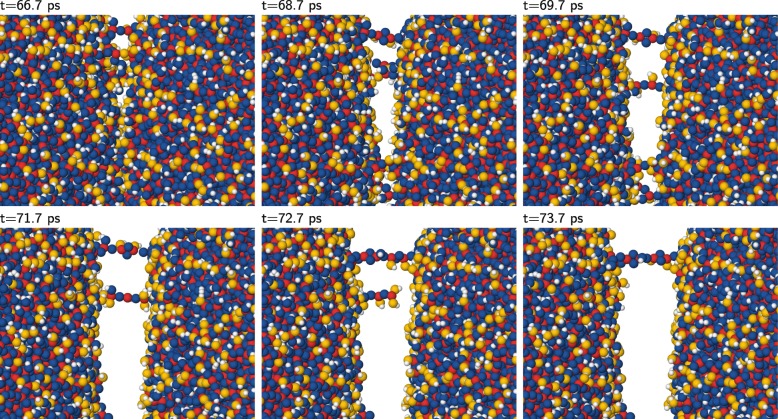
Series of snapshots. Series of snapshots—times of 66.7–73.7 ps after the collision—showing the formation of temporary filaments between two NPs of radius *R*=15 nm after a collision with *v*=75 m/s. Color code as in Fig. 1

The collision dynamics of bare silica NPs is characterized by the formation of a strong adhesive neck between the NPs; during NP separation, this neck is dominated by filaments, i.e., quasi monatomic Si–O–Si–O chains. In the collision of hydroxylated silica NPs, bond passivation largely prevents the formation of such extensive necks. However, occasionally, these necks are also seen between the hydroxylated silica NPs, as we show in Fig. 4 in the form of a time sequence of snapshots zooming into the contact surface of two colliding NPs when they start separating again after the collision. Despite a close contact of the two NPs (Fig. 4a), most regions separate again without leaving much changes on the surfaces behind (Fig. 4b); these unchanged surface patches are regions that are closely packed with passivating H atoms. In selected regions, however, covalent bonds between the two NPs are formed (Fig. 4c), which develop to monatomic filaments (Fig. 4d) in the form of Si–O–Si–O chains that are decorated with OH groups originating from the surface hydroxylation. We note that the Si–O bond strength amounts to 4.70 eV and is hence comparable to the O–H bond strength of 4.77 eV [40], making both the filaments as well as the silanol groups hard to break. Yet, one of these filaments does break during the NP separation while the terminating Si atom catches two OH groups to saturate its bonds (Fig. 4e). However, the second (upper) monatomic chain does not tear (Fig. 4f). We followed the simulation until it was clear that the two NPs were brought together eventually again such that this particular collision was a sticking collision.

## Conclusions

Atomistic simulations of the silica-water interface are non-trivial due to the chemical processes occurring. These lead to a passivation of dangling oxygen and silicon bonds by H ^+^ and OH ^−^ ions, respectively. We use the REAX force field, which allows for bond breaking and formation, to model the passivation of the silica surface. The collision of two hydroxylated silica nanoparticles deviates strongly from the collision of bare silica nanoparticles studied previously. While bare silica NPs stick upon collision for a wide range of collision velocities, hydroxylated nanoparticles already bounce at rather modest velocities. By comparing to the JKR theory of NP collision, this reduction in the bouncing velocity can be attributed to the strong decrease in surface energy effected by the hydroxylation.

Our simulations were performed for NP temperatures of 200 K, far below the temperature, where the hydroxylation layer starts dissolving (460 K) [15, 24]. The temperature chosen by us applies to the asteroid belt. Their collisions between asteroids create a steady-state distribution of NPs (the so-called debris disk [6–8]), where collision velocities of several hundred meters per second are not untypical. Since the bouncing velocity decreases with the NP radius as *R*^−5/6^, see Eq. 2 and Fig. 3, our results are also relevant for larger grains at correspondingly smaller collision velocities.

Future research will aim at extending the present study to silica-ice core-shell systems. Such systems constitute an important species of dust particles in planetary systems beyond the snow line, and their collision physics will be governed by the properties of both the hard silica core and the softer water-ice shell.

## Data Availability

This study does not use data other than have been provided in the text.

## Abbreviations

NP: Nanoparticle
REAX: Reactive force field
LAMMPS: Large-scale atomic/molecular massively parallel simulator
OVITO: Open visualization tool
COR: Coefficient of restitution
